# Factors governing outbreak dynamics in a forest intensively managed for mountain pine beetle

**DOI:** 10.1038/s41598-020-63388-8

**Published:** 2020-05-05

**Authors:** Mélodie Kunegel-Lion, Mark A. Lewis

**Affiliations:** 1 Department of Biological Sciences, University of Alberta, CW 405 Biological Sciences Bldg, Edmonton, AB T6G 2E9 Canada; 2 Department of Mathematical and Statistical Sciences, University of Alberta, 632 CAB, Edmonton, AB T6G 2G1 Canada

**Keywords:** Ecological modelling, Forest ecology

## Abstract

Mountain pine beetle (MPB) outbreaks have caused major economic losses and ecological consequences in North American pine forests. Ecological and environmental factors impacting MPB life-history and stands susceptibility can help with the detection of MPB infested trees and thereby, improve control. Temperatures, water stress, host characteristics, and beetle pressure are among those ecological and environmental factors. They play different roles on MPB population dynamics at the various stages of an outbreak and these roles can be affected by intensive management. However, to make detailed connections between ecological and environmental variables and MPB outbreak phases, a deeper quantitative analysis on local scales is needed. Here, we used logistic regressions on a highly-detailed and georeferenced data set to determine the factors driving MPB infestations for the different phases of the current isolated MPB outbreak in Cypress Hills. While we showed that the roles of ecological and environmental factors in a forest intensively controlled for MPB are consistent with the literature for uncontrolled forests, we determined how these factors shifted through onset, peak, and collapse phases of the intensively controlled forest. MPB presence mostly depends on nearby beetle pressure, notably for the outbreak peak. However additional weather and host variables are necessary to achieve high predictive ability for MPB outbreak locations. Our results can help managers make appropriate decisions on where and how to focus their effort, depending on which phase the outbreak is in.

## Introduction

The mountain pine beetle (MPB; *Dendroctonus ponderosae*, Hopkins 1902, Coleoptera: Curculionidae, Scolytinae) epidemic behaviour in North American pine forests is causing massive ecological consequences and losses to the timber industry^1^ as well as threatening cultural and tourism activities^2^. As a consequence, MPB outbreaks are actively monitored and heavily controlled in Canadian pine forests^3,4^. Managers face several challenges related to detection and control. An efficient control is direct, early, aggressive, and continuous until the outbreak is suppressed^5^. To be able to implement such control, managers need to have efficient detection methods. Detection could be improved by including different ecological and environmental factors depending on the population phase. In turn, the relevant ecological and environmental factors at each population phase are susceptible to be affected by intensive control.

**Figure 1:**
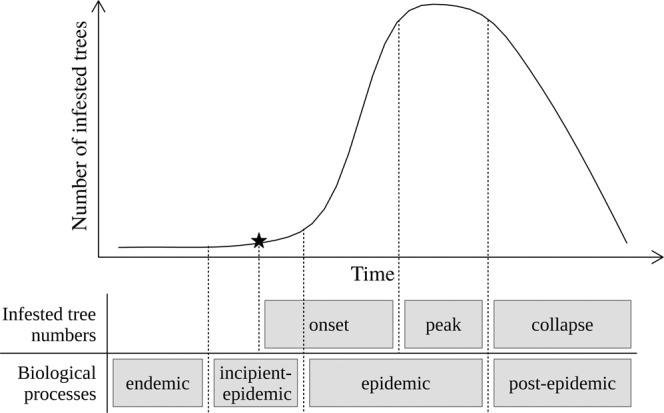
Representation of the biological processes and infested tree numbers approaches to categorizing MPB population phases. The star represents the first detection of the MPB outbreak. The endemic phase has less than one mass-attacked tree per stand.

From the perspective of the biology of MPB, four major phases have been described: endemic, incipient-epidemic, epidemic, and post-epidemic^6^. MPB populations in one of the last three phases form an outbreak. The transition between endemic phase and outbreak depends on population size. Endemic populations have low number of individuals and they attack weak or stressed pines typically with the help of other bark and woodboring beetles. When a MPB population has enough individuals to overcome the defences of large and healthy trees on their own, the population transitions from an endemic phase to an outbreak. Managers, tracking the rise and fall in numbers of infested trees may not easily be able to identify the endemic or early incipient-epidemic phase. This leads to an alternative categorization based on infested tree numbers, which we describe here and use in this paper: onset (increasing number of infested trees; typically late incipient-epidemic and early epidemic), peak (high and constant number of infested trees; late epidemic) and collapse (decreasing number of infested trees; post-epidemic). See Fig. 1 for a representation of the two approaches to categorizing MPB outbreaks.

MPB typically have a one-year life cycle^7^. In the summer, beetles drill galleries under the bark of host trees to lay their eggs^8^. Over the fall, the eggs become larvae. In the spring, individuals resume their development to emerge as adults in the summer. Adults do not usually survive the winter although there is some evidence in northwestern US for overwintering adults^9^. The pine hosts are typically killed by the MPB development process and their crowns fade to a red colour within one year after the attack. The MPB range is widespread in north America, covering north-west United States and western Canada.

At the beginning of an outbreak, *i.e*. the incipient-epidemic phase, spread is slow and infestations are limited to single trees. Then, as population size increases and single-tree infestations become patches containing multiple infested trees, the MPB population enters the epidemic phase. The post-epidemic phase is characterized by population decline in MPB. An outbreak usually lasts several years if sufficient host pines are present, with an average of approximately 10 years in British Columbia^10^.

Commonly, red-top trees, which are the distinct dead pines infested by MPB individuals from the previous year, are used to estimate the presence of new infestations in the area^3,4^. Additionally, the locations of infested trees can be georeferenced to characteristic ecological and environmental factors. However, these factors have different roles on MPB population dynamics at the various stages of an outbreak^11,12^. Moreover, intensive MPB control is likely to impact spatio-temporal patterns of infestations^13^, which might, in turn, affect the significance of ecological and environmental factors in the different outbreak phases. Understanding these roles provides an opportunity to improve detection methods through a systematic evaluation of cues from ecological and environmental factors.

Much of our understanding of the ecological and environmental factors governing MPB outbreaks come from the conceptual and observational work synthesized by Safranyik, Carroll and coworkers^8,10^. Those factors consist of host tree properties, beetle pressure, and weather factors. Host tree properties affect susceptibility to infestation. Beetle pressure provides an indication of the size and proximity of nearby infestations that could spread to new host trees. Weather factors impact the details of life-history and environmental stress of both the beetles and the trees. Collectively, these factors determine the outbreak level and duration.

Host tree abundance, resistance, and size impact MPB infestation differentially, depending on the phase of an outbreak^10^. Indeed, an MPB endemic population first needs sufficient small and weak/stressed trees in order to increase the population size to outbreak levels and attack larger and healthier trees^10,14^. MPB population decline happens in the post-epidemic phase when the number of susceptible pines decreases and MPB switches back to weaker and smaller trees. This decline in the epidemic that is associated with the reduction in the susceptible population is a common feature characterizing epidemic processes^15^.

Beetle pressure is essential for new MPB infestations at all outbreak phases^12^. It describes the source of a new beetle generation. Outbreak onset relies on local endemic population increase and/or contributions from outside sources via dispersal whereas established outbreaks relies on adjacent sources^10,11,14^.

Among weather factors, temperatures have the greatest impact on MPB population life-history^16^. Warm winter temperatures help MPB individuals in endemic phase to transition to an outbreak by allowing them to survive the cold season in greater proportions, thereby possibly increasing their population size to outbreak levels^11,12,17,18^. However, cold snaps in fall or early spring, or generally lower winter temperatures, can lead to outbreak collapse^10,19^. Beetles in the egg stages are also extremely vulnerable to temperatures^20^ and their exposure to mortality-inducing temperatures depends on the timing of adult emergence and oviposition^21^. Therefore, temperatures over the year are good indicators of beetle development rate and timing. Individuals need a minimum of 833 degree-days above 5.5 C to complete their life-cycle^22^ and high temperatures during flight periods increase attack success rate by increasing spatial synchrony^11^. However, excessively high temperatures during the summer can decrease emergence rate as well as dispersal success^8,23^. In summary, warm temperatures are crucial to MPB development but this positive effect on MPB infestation can be counteracted when temperatures become too high for successful emergence and dispersal.

Soil moisture is also an essential factor governing MPB populations. Water deficit lowers pine defenses against MPB attacks^24^. These defences consist of the exudation of toxin resin containing phytochemicals that prevent MPB from attracting conspecifics and inhibit the formation of galleries and oviposition^25,26^. Therefore, drought can help MPB endemic populations successfully attack sufficient trees to increase their population size to outbreak levels^10,11^. However, it is also necessary to have abundant vigorous trees for a successful outbreak^10,14^ and tree vigour can be reduced by drought^27^. Nonetheless, drought may not be sufficient to decrease vigour levels to the point of a suppressed outbreak. For example, entire outbreaks in western US were exposed to drought^18^. Therefore, the timing and intensity of drought can either help or hinder MPB populations.

The combination of observations and data analyses helps picture how ecological and environmental factors change in each outbreak phase. However, a deeper quantitative analysis specifically focusing on this question is required with detailed connections between models and data via statistical inference. Such analyses exist, but, to date, have employed large spatial scales, typically with different sub-regions in different outbreak stages^12,18^. Moreover, management changes the pattern of MPB spread^13^. Therefore, the ecological and environmental factors explaining MPB presence could be affected by intensive control for each outbreak phase. To the best of our knowledge, there has been no local-scale statistical analysis of ecological and environmental factors governing an intensively controlled MPB outbreak, from onset to collapse, where the population is relatively synchronized across the study site. The recent Cypress Hills MPB outbreak in Saskatchewan provides a unique opportunity to do this very thing. Located far from the main lodgepole pine range, the Cypress Hills MPB infestation is isolated from other outbreaks. The Cypress Hills park spatial scale (184 km^2^) is such that the MPB population has been relatively synchronized spatially. The data set is very high quality as the region was completely censused for MPB infestation yearly from the onset in 2006 up to the current decrease in 2018. This provides a unique opportunity to follow a single outbreak in a fixed location at a small spatial scale, and to perform a comprehensive statistical analysis of ecological and environmental factors influencing each outbreak phase.

Our study aims to 1) develop a model to determine the local ecological and environmental factors driving MPB presence for the different phases of an outbreak (onset, peak, and collapse) in a forest intensively managed for MPB, 2) assess the degree to which the models predict MPB presence for each outbreak phase, and 3) show how selected factors have differing impacts on MPB presence depending on the outbreak phase. For each phase, we hypothesized that MPB presence depends on a combination of weather, beetle pressure, control, and host-related variables. We model those relationships using logistic regressions during the onset, peak, and collapse phases of one MPB outbreak studied in the Cypress Hills interprovincial park in Saskatchewan, Canada.

## Methods

### Study area and data

**Figure 2:**
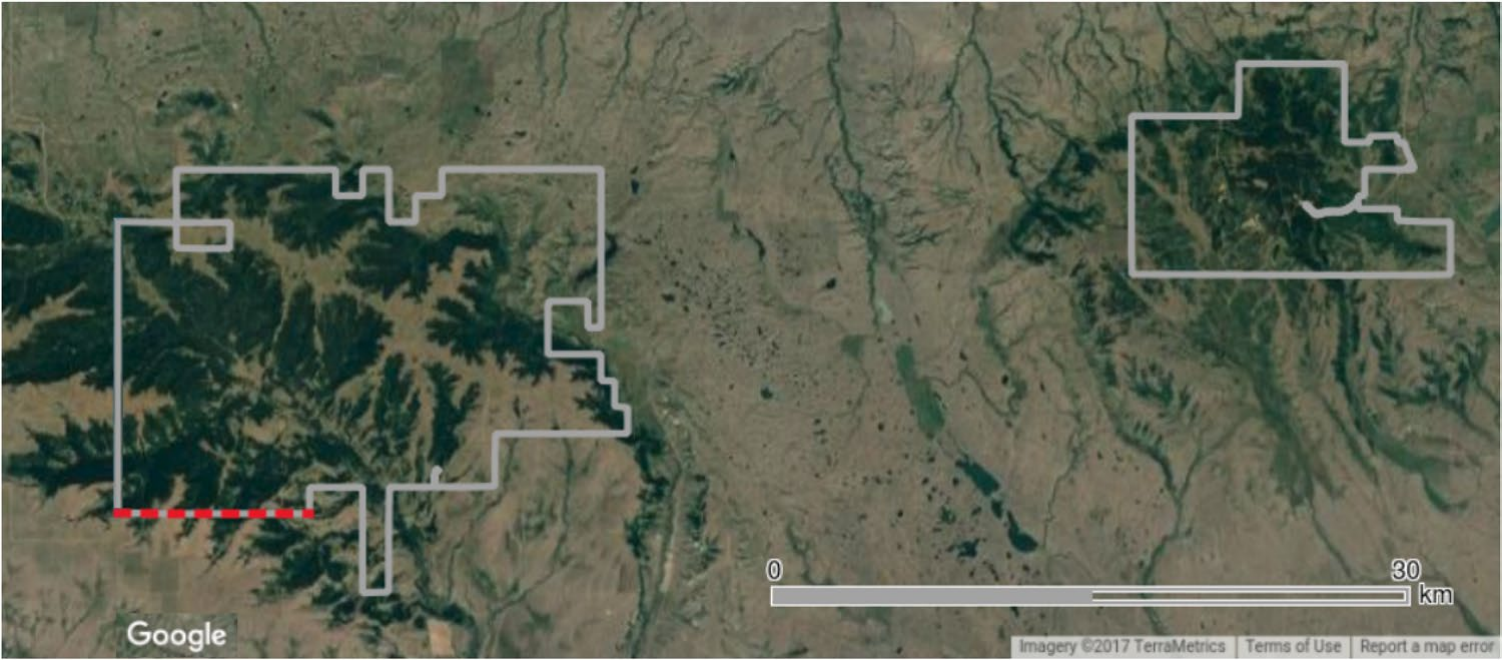
Cypress Hills park boundaries in Saskatchewan (grey). The dashed red line represents the park border close to outside infestations in the South. Based on Kunegel-Lion^62^.

We use data from the Saskatchewan portion of the Cypress Hills interprovincial park, located at the border between the provinces of Alberta and Saskatchewan, Canada. This portion of the park is separated in two blocks 20 km apart (Fig. 2). The main MPB host tree in this area is the lodgepole pine (*Pinus contorta*, Dougl. ex Loud. var. *contorta* Engelm). MPB infestation data and ecological and environmental covariates from this region provide an opportunity to connect local factors to outbreak phases. In addition to the infested trees within the park limits, there were infestations just outside the park in the south (Fig. 2). These infestations were not recorded nor managed and they were slightly ahead of time compared to the park infestations. The Forest Service Branch of the Saskatchewan Ministry of Environment has implemented a “zero-tolerance” policy as of 2006, designed to catch and control as many newly infested trees in the park as possible. Details of this policy are described in Kunegel-Lion *et al*.^28^.

**Figure 3:**
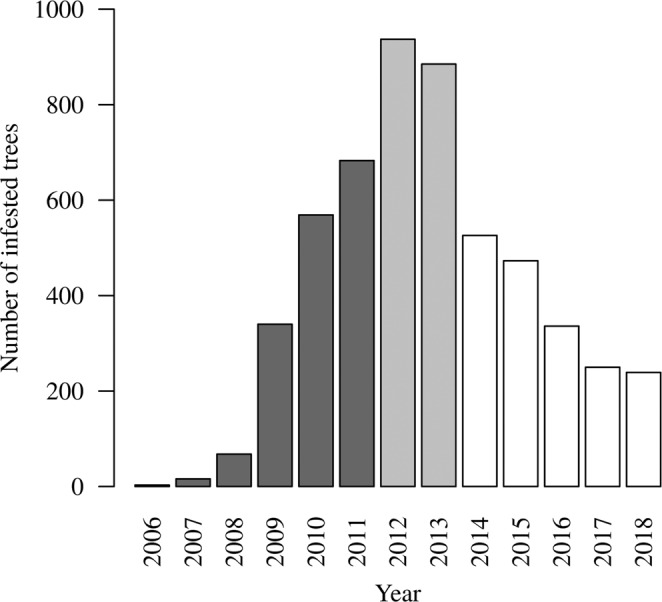
Total number of infested trees over time. The darker grey represents the outbreak onset. The grey represents the outbreak peak. The white represents the outbreak collapse.

The ecological and environmental covariates and the infestation response values were distributed discretely in space and time. We applied a grid of 18 317 cells of size 100 × 100 meters to the Cypress Hills park extent. This cell size was chosen to match the size of the management surveys. The fact that a cell’s area (10 000 m^2^) and a search plot’s area (7 854 m^2^) are the same order of magnitude make the analogy between grid cell and survey plot possible. For each combination of cell and year, the observation consisted of a set of ecological and environmental covariates plus the response variable. The response variable was the presence/absence of MPB derived from the presence/absence of infested trees in each cell of the grid based on data from the Forest Service ground survey^4,28^. From these Forest Service surveys, we obtained the locations of infestations controlled by managers and we deduced which trees had been infested in the previous year using the red-top trees. We divided these data into outbreak phases and trained and validated the models separately for each phase. The outbreak onset lasted from 2006 to 2011, the outbreak peak from 2012 to 2013, and the outbreak collapse from 2014 to 2018 (Fig. 3).

We chose the ecological and environmental covariates to represent as much as possible each of the processes described in the introduction (Table 1). Description of these variables is available in Kunegel-Lion *et al*.^28^.

**Table 1:** Description and range of the variables used in the logistic regressions.

Name	Description	Range	Unit
T_max_	Highest maximum daily temperature during July and August	26.2–38.2	°C
T_min_	Lowest minimum daily temperature during July and August	−8.2–6.1	°C
SMI	Soil moisture index^63^ estimates soil water availability for tree growth using temperature and precipitation	36.4–97.3	mm
CT	Cold tolerance^64^ estimates the probability of larva survival over the winter using temperature	24.1–86.0	%
Peak	MPB emergence peak (derived from Bleiker and Van Hezewijk’s model 1^51^) estimates when 50% of the beetles have emerged from cumulative degree-days above 2 °C starting on May 30th (Julian day 150)	205–232	Julian day
Cover	Coverage of *Pinus albicaulis* (whitebark pine), *Pinus banksiana* (jack pine) and *Pinus contorta* (includes subspecies lodgepole pine and shore pine)	0.0–97.2	%
Height	Height of the dominant tree species in the cell when the pine cover is greater than 50%	0.0–51.5	m
Age	Age of the dominant tree species in the cell when the pine cover is greater than 50%	0.0–200.3	year
	Previous-year controlled MPB infestation level in a 3-cell radius around each location		
I_*c*_	I_*c*_ = number of infested cells with all trees controlled at the same location + 0.5 ×number of infested cells with all trees controlled in radius 1 + 0.25 ×number of infested cells with all trees controlled in radius 2 + 0.125× number of infested cells with all trees controlled in radius 3 (Fig. 4)	0.00–5.00	—
	Previous-year uncontrolled MPB infestation level in a 3-cell radius around each location		
I_*u*_	I_*u*_ = number of infested cells with uncontrolled trees at the same location + 0.5× number of infested cells with uncontrolled trees in radius 1 + 0.25 ×number of infested cells with uncontrolled trees in radius 2 + 0.125× number of infested cells with uncontrolled trees in radius 3 (Fig. 4)	0.00–4.25	—
Dist	Distance to the park southern border close to external infestations (Fig. 2)	67.6–36151.6	m
N	Northerness: spatial property of a slope to face North	−1–1	—
E	Easterness: spatial property of a slope to face East	−1–1	—

**Figure 4:**
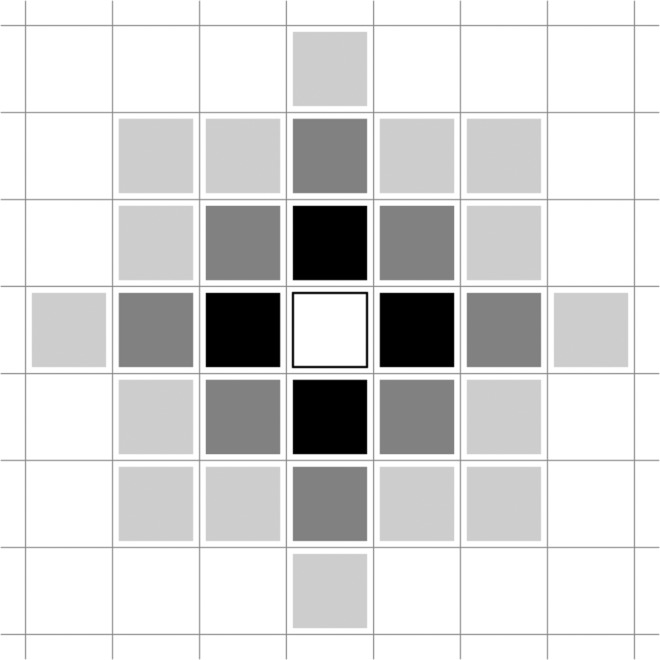
Representation of the adjacent cells taken into account in the covariates (cf. Table 1). White: focus cell; dark grey: 4 adjacent cells (radius 1); medium grey: next 8 adjacent cells (radius 2); light grey: next 16 adjacent cells (radius 3). Based on Kunegel-Lion^62^.

For a given year, infested locations can be divided into uncontrolled infestation (I_*u*_) and controlled infestation (I_*c*_). Details of these variables are given in Table 1 and Fig. 4. However, some infested trees inevitably remained undetected and these were identified as red-tops in the following year. Beetles from these uncontrolled infestations can disperse short distances within and between cells, and thus provide a source for new infestations. We refer to this as nearby beetle pressure. The MPB presence two years prior to the observation is not included as MPB is generally univoltine^29^, so we assume that an infested tree can only be a source of beetle for the following year and not the years after that. We also included the distance to the park southern border, which was close to external infestations not managed by the Forest Service and potential sources of MPB. Finally, it is possible to calculate the total infestation by adding the uncontrolled and controlled infestations.

### Data analysis

In this analysis, we select, for each outbreak phase, the weather, vegetation, topography, and beetle and host-related variables explaining MPB presence in a forest intensively controlled for MPB and show whether the selected variables have a different impact on MPB presence depending on the outbreak phase.

To select the relevant variables explaining MPB presence, we used a logistic regression where the probability of MPB presence $\pi (\underline{\beta })$ depend on parameters ${\beta }_{i}$ and ecological and environmental covariates ${X}_{i}$, described in the previous section, as defined by

1 $$\pi (\underline{\beta })=\frac{{e}^{{\beta }_{0}+\sum {\beta }_{i}{X}_{i}}}{1+{e}^{{\beta }_{0}+\sum {\beta }_{i}{X}_{i}}}\mathrm{}.$$

We trained the logistic regressions on each phase separately using the train function of the R package caret^30,31^.

For each outbreak phase, we implemented a multiple working hypothesis approach. We used the exhaustive enumeration of subsets method^32^. This method compares all possible combinations of the covariates and selects the best models among the ones sharing the same number of covariates. We selected the best model overall and the best model per number of covariates using the Bayesian Information Criterion (BIC)^33^. With our goal of determining which factors are associated with MPB infestation at each outbreak phase, we chose the BIC over the Akaike Information Criterion (AIC)^34^ for model comparison. This is because BIC is better indicator of the “true” model whereas AIC is more suited to determine which models should be used for predictions^35–37^. Additionally, our number of observations was very large (238121 observations) compared to the parameter space (11 parameters) which also favours the BIC over the AIC. As with the AIC index, a low BIC means a good trade-off between the goodness of fit of the model and model complexity. Two models with a BIC difference less or equal to 2 are considered indistinguishable whereas a BIC difference of 8 or more provides strong evidence for the model with lower BIC^38,39^.

To be able to differentiate the effect of each covariate, we categorized them into weather (T_max_, T_min_, SMI, CT, and Peak), vegetation (Cover, Age, and Height), topography (Dist, N, and E), or beetle-related (I_*c*_ and I_*u*_) variables and we removed, within each category, highly correlated covariates ($|\rho |\, > \,0.6$) but one from our analysis. Therefore, among the weather variables, we did not include the maximum temperature in the analysis as it was correlated with the soil moisture index ($\rho =-\,0.69$) and with the emergence peak ($\rho =-\,0.76$) over the entire time period. Indeed, these three indices are all derived from daily temperatures. As might be expected, among the vegetation-related variables, pine height and age were also correlated ($\rho =0.95$). Therefore, of these two, we only kept pine height in our analyses. Note that, in the beetle-related variables, the correlation coefficient for the controlled and uncontrolled infestations is 0.33, therefore the impact of these covariates on the infestation should be possible to differentiate. In addition, *a posteriori*, we did not keep models with poorly estimated coefficients due to multicollinearity, *i.e*. models with a maximum variance inflation factor (VIF) greater than 10^40,41^.

To assess the performance of the selected models, we performed cross-validation with folds defined by year. For each outbreak phase, the cross-validation process works as follow: 1) the models are trained on all years of their respective outbreak phase but one, 2) the accuracy of the models is tested on the remaining year of the outbreak phase (=test set) using the area under the receiver operating characteristic curve (AUROC)^42,43^ and the area under the precision-recall curve (AUPR)^44,45^, and 3) steps 1 and 2 are repeated with a different year held out for test until all years have been tested. The AUROC and AUPR indices are then averaged over the folds.

A receiver operating characteristic (ROC) curve^42^ depicts, for a range of probability thresholds, the true positive rate (or 1 - false negative rate, also referred to as sensitivity or recall) against the false positive rate (also referred to as 1 - specificity). A precision-recall curve^44^ depicts, for a range of probability thresholds, the proportion of true positives amongst the positive predictions (also referred to as precision or positive predictive value) against the true positive rate (sensitivity/recall). For the reader’s convenience, more details on how to calculate these indices are given in Appendix A.

A high AUROC or AUPR ($0\le AUROC/AUPR\le 1$) represents a good performance of a binary classifier in terms of correspondence between observed and predicted values. A null model has an AUROC of 0.5 and a AUPR value equals to the proportion of positive outcomes in the data. The precision-recall curve is more informative than the ROC curve for imbalanced data sets^45,46^ which is the case here as the rate of 0 to 1 in our three data sets is between 40:1 and 95:1.

To show whether the selected covariates have a different impact depending on the outbreak phase, we compared the order of importance of the standardized estimates ${\beta }_{i}$. Within a model, a large negative or positive ${\beta }_{i}$ has, respectively, a large negative or positive impact on MPB presence whereas a small ${\beta }_{i}$ has a small impact on MPB presence.

## Results

For the outbreak onset, the best model used seven covariates: northerness, soil moisture index, distance to the infested border, emergence peak, pine cover, and controlled and uncontrolled infestations (BIC = 8626.9; Table 2). However, the model with the covariate pine height instead of pine cover had Δ BIC $\mathrm{ < 8}$ which implies that pine height and pine cover could be interchanged.

**Table 2:** Comparison of the models’ BIC, AUROC, and AUPR for the outbreak onset.

Size	Selected variables	VIF_*max*_	BIC	Δ BIC	AUROC	AUPR
0	*null*		12705.2	4078.3	0.500	0.010
1	I_*u*_	1.0	10121.8	1495.0	0.650	0.154
2	Peak, I_*u*_	1.0	9317.5	690.6	0.770	0.142
3	Peak, I_*u*_, I_*c*_	1.0	8787.9	161.0	0.786	0.172
4	Dist, Peak, I_*u*_, I_*c*_	1.2	8718.7	91.8	0.866	0.180
5	SMI, Dist, Peak, I_*u*_, I_*c*_	1.6	8675.3	48.4	0.705	0.179
6	SMI, Dist, Peak, Cover, I_*u*_, I_*c*_	1.6	8644.6	17.7	0.731	0.176
**7**	**N, SMI, Dist, Peak, Cover,I**_***u***_**, I**_***c***_	**1.6**	**8626.9**	**0.0**	**0.739**	**0.174**
7	N, SMI, Dist, Peak, Height, I_*u*_, I_*c*_	1.6	8633.4	6.5	0.731	0.174
**8**	**N, SMI, Dist, Peak, Cover, Height, I**_***u***_**, I**_***c***_	**1.6**	**8628.3**	**1.4**	**0.741**	**0.173**
8	N, T_min_, SMI, Dist, Peak, Cover, I_*u*_, I_*c*_	2.3	8630.1	3.2	0.743	0.175
9	N, T_min_, SMI, Dist, Peak, Cover, Height, I_*u*_, I_*c*_	2.3	8631.4	4.5	0.745	0.174
10	N, E, T_min_, SMI, Dist, Peak, Cover, Height, I_*u*_, I_*c*_	2.3	8639.8	12.9	0.751	0.173
11	N, E, T_min_, SMI, CT, Dist, Peak, Cover, Height, I_*u*_, I_*c*_	2.4	8650.6	23.7	0.751	0.172

All models are compared to the one with the lowest BIC using ΔBIC. For each number of variables, we show the best model and competing models with a difference of BIC ≤8. The model in bold is the one selected from the ΔBIC ≤ 2. “AUROC” stands for the area under the ROC curve, “AUPR” stands for the area under the precision-recall curve.

For the peak of the outbreak, the best model used seven covariates: easterness, minimum temperature in summer, cold tolerance, emergence peak, pine height, and controlled and uncontrolled infestations (BIC = 5480.4; Table 3). However, the model without easterness gave a Δ BIC $\mathrm{ < 8}$, casting doubt on the importance of this index on MPB presence for the peak.

**Table 3:** Comparison of the models’ BIC, AUROC, and AUPR for the outbreak peak.

Size	Selected variables	VIF*max*	BIC	Δ BIC	AUROC	AUPR
0	*null*		8341.5	2861.1	0.500	0.024
1	I_*u*_	1.0	5557.7	77.3	0.879	0.436
2	Height, I_*u*_	1.0	5540.7	60.3	0.902	0.430
3	Height, I_*u*_, I_*c*_	1.4	5520.4	40.0	0.907	0.427
4	E, Height, I_*u*_, I_*c*_	1.4	5507.8	27.4	0.907	0.429
5	CT, Peak, Height, I_*u*_, I_*c*_	4.1	5497.4	17.0	0.912	0.425
6	T_min_, CT, Peak, Height, I_*u*_, I_*c*_	6.9	5485.5	5.1	0.886	0.400
**7**	**E, T**_**min**_**, CT, Peak, Height, I**_***u***_**, I**_***c***_	**6.9**	**5480.4**	**0.0**	**0.890**	**0.404**
8	E, T_min_, CT, Peak, Cover, Height, I_*u*_, I_*c*_	7.2	5484.8	4.4	0.890	0.402
9	E, T_min_, CT, Dist, Peak, Cover, Height, I_*u*_, I_*c*_	7.1	5494.9	14.5	0.889	0.401
10	N, E, T_min_, CT, Dist, Peak, Cover, Height, I_*u*_, I_*c*_	7.1	5505.4	25.0	0.891	0.404

All models are compared to the one with the lowest BIC using ΔBIC. For each number of variables, we show the best model and competing models with a difference of BIC ≤8. The models in bold are the ones selected from the ΔBIC ≤ 2. “AUROC” stands for the area under the ROC curve, “AUPR” stands for the area under the precision-recall curve.

The best model for the outbreak collapse used seven covariates: minimum temperature in summer, soil moisture index, cold tolerance, distance to the infested border, emergence peak, and controlled and uncontrolled infestations (BIC = 7448.5; Table 4).

**Table 4:** Comparison of the models’ BIC, AUROC, and AUPR for the outbreak collapse.

Size	Selected variables	VIF_*max*_	BIC	Δ BIC	AUROC	AUPR
0	*null*		11071.0	3622.5	0.500	0.011
1	I_*u*_	1.0	8182.2	733.7	0.864	0.264
2	I_*u*_, I_*c*_	1.0	7498.5	50.0	0.929	0.306
3	SMI, I_*u*_, I_*c*_	1.0	7468.9	20.3	0.935	0.305
4	SMI, Dist, I_*u*_, I_*c*_	1.1	7458.0	9.5	0.937	0.305
5	SMI, Dist, Peak, I_*u*_, I_*c*_	1.4	7462.7	14.2	0.936	0.305
6	T_min_, SMI, CT, Dist, I_*u*_, I_*c*_	3.2	7461.2	12.7	0.935	0.305
**7**	**T**_**min**_**, SMI, CT, Dist, Peak**, I_*u*_, I_*c*_	**3.3**	**7448.5**	**0.0**	**0.934**	**0.303**
8	T_min_, SMI, CT, Dist, Peak, Cover, I_*u*_, I_*c*_	3.3	7454.3	5.8	0.935	0.301
9	E, T_min_, SMI, CT, Dist, Peak, Cover, I_*u*_, I_*c*_	3.3	7464.2	15.6	0.934	0.300
10	N, E, T_min_, SMI, CT, Dist, Peak, Cover, I_*u*_, I_*c*_	3.3	7474.8	26.3	0.934	0.299
11	N, E, T_min_, SMI, CT, Dist, Peak, Cover, Height, I_*u*_, I_*c*_	3.3	7485.6	37.1	0.933	0.299

All models are compared to the one with the lowest BIC using ΔBIC. For each number of variables, we show the best model and competing models with a difference of BIC ≤8. The model in bold is the one selected from the ΔBIC ≤ 2. “AUROC” stands for the area under the ROC curve, “AUPR” stands for the area under the precision-recall curve.

For each phase, the selected models have high AUROC indicating a high level of predictive ability (${{\rm{AUROC}}}_{best,onset}=0.739$, ${{\rm{AUROC}}}_{best,peak}=0.890$, and ${{\rm{AUROC}}}_{best,collapse}=0.934$; Tables 2 to 4). Compared to the null models, the AUPR values are consistent with the higher AUROC values (${{\rm{AUPR}}}_{best,onset}=0.174$ with ${{\rm{AUPR}}}_{null,onset}=0.010$, ${{\rm{AUPR}}}_{best,peak}=0.404$ with ${{\rm{AUPR}}}_{null,peak}=0.024$, ${{\rm{AUPR}}}_{best,collapse}=0.303$ with ${{\rm{AUPR}}}_{null,collapse}=0.011$; Tables 2 to 4). The relatively high AUPR values show that the models predict well MPB presence without potentially wasting too much management resources on false alerts, which are incorrectly-predicted MPB presence.

For the three outbreak phases, the model including only information about nearby beetle pressure (I_*u*_) is the best model among the ones with one covariate. Compared to the overall best model of each phase, the model including only nearby beetle pressure is predicting MPB presence with a lower accuracy (${{\rm{AUROC}}}_{{I}_{u},onset}=0.650$, ${{\rm{AUROC}}}_{{I}_{u},peak}=0.879$, and ${{\rm{AUROC}}}_{{I}_{u},collapse}=0.864$; Tables 2 to 4). The AUPR values are consistent with these AUROC values except for the outbreak peak where the AUPR value is higher for the model with only nearby beetle pressure (${{\rm{AUPR}}}_{{I}_{u},peak}=0.436$).

**Figure 5:**
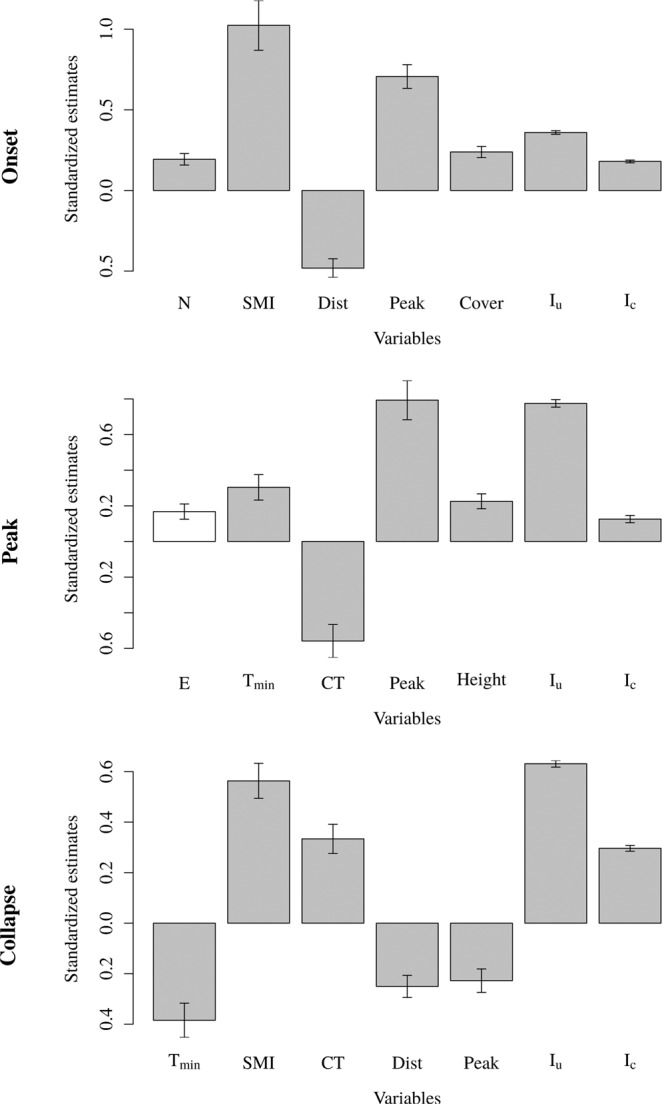
Standardized estimates (± standard error) for each selected model by outbreak phases. Variables in white have weak evidence from ΔBIC (see Tables 2 to 4).

For the outbreak onset, the order of the covariates by importance (absolute standardized estimates) is: soil moisture index, emergence peak, distance to the infested border, uncontrolled infestations, pine cover, northerness, controlled infestations (Fig. 5). The order and the selected covariates differ from the peak: emergence peak, uncontrolled infestations, cold tolerance, minimum temperature in summer, pine height, easterness, controlled infestations, and the collapse: uncontrolled infestations, soil moisture index, minimum temperature in summer, cold tolerance, controlled infestations, distance to the infested border, emergence peak (Fig. 5).

Some covariates have a negative impact on MPB presence. A larger distance to the infested border decreases the probability of infestation in a cell (Fig. 5). Other covariates have a positive impact on MPB presence. A larger nearby controlled or uncontrolled infestation, larger soil moisture index, higher pine cover, larger pine height, northerness, or easterness increases the probability of infestation in a cell (Fig. 5). The minimum temperature in summer has both a positive impact of MPB presence at the outbreak peak and a negative impact at the outbreak collapse. The cold tolerance has a negative impact at the outbreak peak and a positive impact at the outbreak collapse. Finally, the emergence peak has a positive impact at the outbreak onset and peak and a negative impact at the outbreak collapse (Fig. 5).

**Figure 6:**
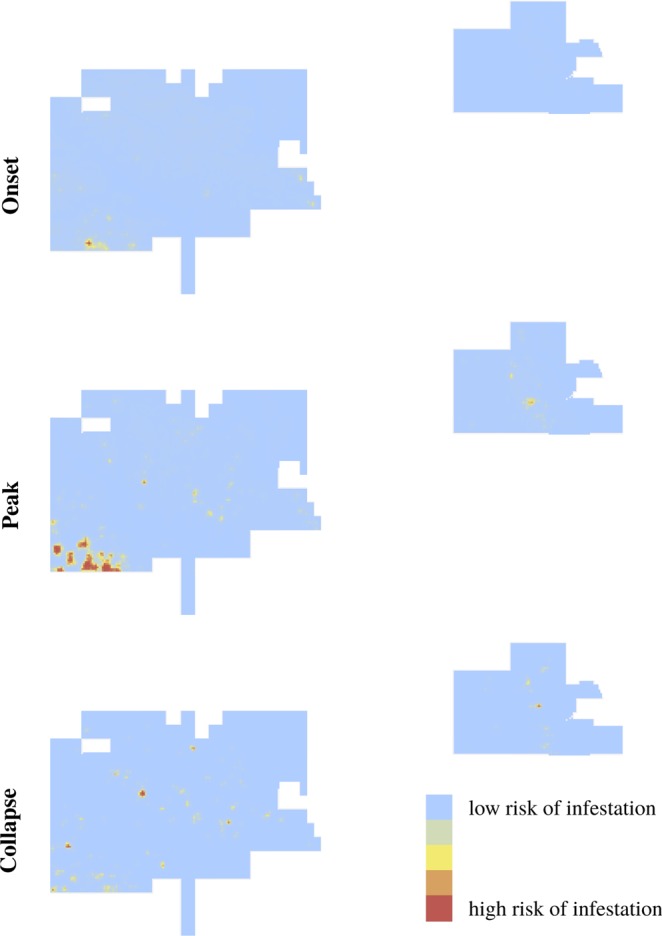
Maps of the predicted infestation probabilities $\pi (\underline{\beta })$ using the parameters from the best model for each outbreak phase. The onset is represented by the year 2009, the peak by the year 2013, and the collapse by the year 2016. For each outbreak phase, the prediction patterns are similar among years. The risk of infestation ranges from low (blue) to high (red).

We can visually characterize the spatial patterns of infestations for each outbreak phase. During the onset, there are few large areas with high infestation risk and they are directly adjacent to the park infested border (Fig. 6). However, other smaller areas at risk are present in the rest of the park. During the peak, more large areas with high risk of infestation arise and they are located nearby previous infestations rather than adjacent to the park infested border (Fig. 6). Note that since the first areas with high infestation risk were close to this border, most areas at risk during the peak are still located in the same general region. During the collapse, the areas with high risk of infestation generally decrease in size but locations similar to the ones during the peak are at risk (Fig. 6).

## Discussion

Our analyses showed how the impact of environmental variables on MPB infestation change with the outbreak phase in a forest managed for MPB. The selected models showed high AUROC values and relatively high AUPR values compared to the null models suggesting, respectively, good predictive abilities overall and good predictions of MPB presence while avoiding false alerts. Therefore most variables driving MPB infestations are likely included in the models.

The selected environmental factors and their impact change depending on the outbreak phase. The source of beetles, represented by the presence of uncontrolled infestations in the neighbourhood and the distance to the infested border, always have a major impact on MPB presence. The importance of beetle pressure and history has also been widely found in previous studies^10–12^. However, the presence of uncontrolled infestations by itself is less influential during the onset. The source of beetles shifts from the infested border during the onset to nearby uncontrolled infested trees as beetles establish themselves in patches at the peak and collapse phases. Perhaps surprisingly, this positive impact on MPB presence was also seen for entirely controlled cells, albeit at a lower level. We interpret this as being correlative but not causal, and arising from the MPB showing a preference for certain environmental conditions. These conditions persist from year-to-year even after the MPB infested trees are controlled. Good management reduces effectively the likeliness of infestation, since controlled infestations always have a lower impact on MPB presence than the presence of uncontrolled trees.

The pine height and cover have a limited impact on MPB presence. Pine height does not explain MPB presence during the outbreak collapse suggesting that beetles are not targeting taller trees at that time. However, MPB presence is higher in locations with taller trees during the outbreak onset and peak. Given the positive relationship between tree diameter and height^47^, this result agrees with the fact that sufficient large and vigorous pines are necessary to sustain high epidemic population size^10^. Northerness and easterness have very little impact on MPB presence although they are selected during the selection process, respectively at the outbreak onset and peak. At the onset, MPB presence is more likely in locations facing north whereas at the peak, MPB presence is more likely in locations facing east. This directionality could be explained by the fact that the dominant winds in Cypress Hills come from the south-west. Indeed, MPB tends to disperse downwind until they encounter an attractive odour which they then follow upwind^48,49^.

The weather factors have a varying impact on MPB presence throughout the outbreak. The minimum temperature over the summer increases the probability of MPB presence during the outbreak peak. Cold temperatures in the summer decrease adult emergence and dispersal and egg survival^20^. However, this factor has a negative relationship with MPB presence during the outbreak collapse. This could be explained by the possibility that, during the collapse, temperatures cold enough to have an impact on beetle emergence and egg survival are not reached. Higher larvae cold tolerance over the winter increases MPB presence during the outbreak collapse. This result is also found in other study^11,12,18^. This relationship is expected as warm winter temperatures help increase beetle population size^19^. However, higher cold tolerance decreases the probability of MPB presence during the outbreak peak. The VIF for the cold tolerance during the outbreak peak is above 5 (another commonly used cutoff^41^) which suggests that this unexpected direction of impact might be the result of a confounding effect. The soil moisture index is having a strong positive impact on MPB presence except during the outbreak peak. This direction of impact is unexpected as water deficit decreases the pines’ ability to defend themselves against MPB^24^. However, soil moisture tends to increase within a year of MPB attacks due to a reduction in evapotranspiration^50^. Since there is a spatio-temporal correlation between locations previously attacked and locations currently attacked by MPB^11^, the relationship between the decrease in evapotranspiration and MPB attacks could essentially create a link between wetter locations and current MPB presence, thereby biasing the soil moisture covariate. During the outbreak onset and peak, MPB presence increases with later estimated emergence peak whereas during the collapse MPB presence increases with earlier estimated emergence peak. Over the entire study period, the estimated peak date ranges from July 24 to August 20 (Julian days 205–232) which is comparable to what was previously observed for mountain pine beetle^8,51^. However, since the flight period model was estimated for north-central Alberta^51^ and not Cypress Hills, the estimated emergence peak date is likely biased. Indeed, MPB development timing changes with latitude^52^.

Overall, MPB presence during the outbreak peak mostly depends on nearby beetle pressure whereas it relies more on additional weather and host conditions for the other outbreak phases. Despite intensive management of the study area, the relevant factors of each phase are mostly in agreement with the literature. Therefore, control is likely not impacting considerably the roles of ecological and environmental variables.

The presence of uncontrolled infestations in the neighbourhood the previous year is consistently a good predictor of MPB presence. Furthermore, we found that this variable is the single most important covariate explaining MPB presence at each outbreak phase. Indeed, including beetle pressure is essential for MPB detection^53^. Using only nearby beetle pressure, as it is mostly the case for the current detection strategy in Cypress Hills, we obtain a poor detection ability (as estimated using the AUROC) at the outbreak onset and a rather satisfactory detection ability at the outbreak peak and collapse. However, the addition of weather and host characteristics improves our detection ability. Susceptibility indices for MPB typically focus on detailed host and stand characteristics^54–57^. This is because managers can actively modify stand characteristics in most cases. Therefore, knowing the susceptibility of their stands give them the option of using preventive measures against MPB^58^. However, if the focus is detection and not prevention, adding variables that managers have no control on, like weather variables, increases the predictive accuracy.

A limitation of this work comes from the fact that we are working with presence/absence in cells and not actual numbers of infested trees or beetles. The number of infested trees is a good proxy for the beetle population size^5^. Our use of presence/absence instead of number of infested trees does, however, allow us to deal with the issue that a small part of the data is expressed as infested zones and not actual tree locations.

Some ecological factors influencing MPB infestations, such as predators and competitors, were not available to us and therefore were not included in this analysis. These factors are not as readily and broadly available as weather variables, and thus are often not included in analyses. Other factors linked to host and stand characteristics, such as pine cover and height, were only available for a couple of years within our study period. Therefore, they were largely estimated. However, future work should focus on gathering such data and analyzing the impact of MPB predators and competitors, along with stand characteristics, on MPB location^59–61^.

Finally, when determining the outbreak phases, we considered the outbreak status in Cypress Hills as a whole instead of differentiating the status of each cell. For example, some cells could be newly infested during the outbreak peak or collapse. However, since the study area is small, it makes sense to see the outbreak as a whole as factors usually have larger yearly variations than within-year variations.

To conclude, the impact of weather, vegetation, and beetle or host-related factors on MPB infestations were shown to vary in a clear, ecologically interpretable manner during an outbreak. This gives managers guidance regarding which stands to focus on for an efficient control. For example, they could use the risk probability maps to inform survey locations^62^. These results also point out that the predictive ability of models using data from an incomplete outbreak to determine future infestations may be limited. Indeed, with such a change in the factor impacts from an outbreak phase to another, the predictions for a specific phase should be biased if model training is done with data from another phase. However, while the size of impact does change, the direction of impact of most covariates seldom changes as a function of the outbreak phase so this may limit prediction error.

## Supplementary information

Appendix.

## Data Availability

The dataset analyzed in this study is described in Kunegel-Lion *et al*.^28^ and is available from Dryad repository (https://doi.org/10.5061/dryad.70rxwdbt9).
