# Supplementary material for: Factors governing outbreak dynamics in a forest intensively managed for mountain pine beetle
Source: Sci Rep. 2020 May 5;10:7601. doi: 10.1038/s41598-020-63388-8 (PMC7200669; doi:10.1038/s41598-020-63388-8)
Supplement: Supplementary file 1 — Appendix. [file 41598_2020_63388_MOESM1_ESM.pdf]

Appendix to: Factors governing outbreak dynamics in a forest intensively managed for mountain pine beetle

Mélodie Kunegel-Lion[1,*] **and Mark A. Lewis**[1,2]

[1]Department of Biological Sciences, University of Alberta, CW 405 Biological Sciences Bldg, Edmonton, AB T6G 2E9, Canada

[2]Department of Mathematical and Statistical Sciences, University of Alberta, 632 CAB, Edmonton, AB T6G 2G1, Canada

[*]kunegel@ualberta.ca

ABSTRACT

Mountain pine beetle (MPB) outbreaks have caused major economic losses and ecological consequences in North American pine forests. Ecological and environmental factors impacting MPB life-history and stands susceptibility can help with the detection of MPB infested trees and thereby, improve control. Temperatures, water stress, host characteristics, and beetle pressure are among those ecological and environmental factors. They play different roles on MPB population dynamics at the various stages of an outbreak and these roles can be affected by intensive management. However, to make detailed connections between ecological and environmental variables and MPB outbreak phases, a deeper quantitative analysis on local scales is needed. Here, we used logistic regressions on a highly-detailed and georeferenced data set to determine the factors driving MPB infestations for the different phases of the current isolated MPB outbreak in Cypress Hills. While we showed that the roles of ecological and environmental factors in a forest intensively controlled for MPB are consistent with the literature for uncontrolled forests, we determined how these factors shifted through onset, peak, and collapse phases of the intensively controlled forest. MPB presence mostly depends on nearby beetle pressure, notably for the outbreak peak. However additional weather and host variables are necessary to achieve high predictive ability for MPB outbreak locations. Our results can help managers make appropriate decisions on where and how to focus their effort, depending on which phase the outbreak is in.

APPENDIX A: True and false, positive and negative rates

A true positive (TP) is an observed presence that is also a predicted presence whereas a false negative (FN) is an observed presence that is also a predicted absence. A true negative (TN) is an observed absence that is also a predicted absence whereas a false positive (FP) is an observed absence that is also a predicted presence (Table A1).

Table A1. Confusion matrix displaying the number of observations by observed and predicted outcome.

		Observed	
		Absence	Presence
Predicted	Absence	TN	FN
	Presence	FP	TP

A classifier gives as results the probability of having a presence for each observation; therefore, we determine the number of predicted presence or absence using a probability threshold. For example, setting the threshold to 0.5 would mean that if the probability of having MPB presence is higher than 0.5, we would consider it as a predicted presence and if it were below 0.5, we would consider it an absence. A different probability threshold would then give different number of true positives, false positives, true negatives and false negatives.

A receiver operating characteristic (ROC) curve[1] depicts, for a range of probability thresholds, the true positive rate (or 1 - false negative rate, also referred to as sensitivity or recall) against the false positive rate (also referred to as 1 - specificity). The true positive rate (TPR) is calculated following the equation: $\text{TPR} = \frac{\text{TP}}{\text{TP+FN}}$. The false positive rate (FPR) is calculated following the equation: $\text{FPR} = \frac{\text{FP}}{\text{FP+TN}}$.

A precision-recall curve[2] depicts, for a range of probability thresholds, the proportion of true positives among the positive predictions (also referred to as precision or positive predictive value) against the true positive rate (sensitivity/recall). The precision is calculated following the equation: $\text{precision} = \frac{\text{TP}}{\text{TP+FP}}$.

References

1. Metz, C. E. Basic principles of ROC analysis. *Semin. Nucl. Medicine* **8**, 283–298, DOI: 10.1016/S0001-2998(78)80014-2 (1978).

2. Raghavan, V., Bollmann, P. & Jung, G. S. A Critical Investigation of Recall and Precision As Measures of Retrieval System Performance. *ACM Trans. Inf. Syst.* **7**, 205–229, DOI: 10.1145/65943.65945 (1989).
